# Semi-Supervised Informer for the Compound Fault Diagnosis of Industrial Robots

**DOI:** 10.3390/s24123732

**Published:** 2024-06-08

**Authors:** Chuanhua Deng, Junjie Song, Chong Chen, Tao Wang, Lianglun Cheng

**Affiliations:** Guangdong Provincial Key Laboratory of Cyber-Physical System, Guangdong University of Technology, Guangzhou 510006, China; icefox@outlook.com (C.D.); junjiesong0509@foxmail.com (J.S.); wangtao_cps@gdut.edu.cn (T.W.); llcheng@gdut.edu.cn (L.C.)

**Keywords:** fault diagnosis, deep learning, semi-supervised learning, industrial robots

## Abstract

The increasing deployment of industrial robots in manufacturing requires accurate fault diagnosis. Online monitoring data typically consist of a large volume of unlabeled data and a small quantity of labeled data. Conventional intelligent diagnosis methods heavily rely on supervised learning with abundant labeled data. To address this issue, this paper presents a semi-supervised Informer algorithm for fault diagnosis modeling, leveraging the Informer model’s long- and short-term memory capabilities and the benefits of semi-supervised learning to handle the diagnosis of a small amount of labeled data alongside a substantial amount of unlabeled data. An experimental study is conducted using real-world industrial robot monitoring data to assess the proposed algorithm’s effectiveness, demonstrating its ability to deliver accurate fault diagnosis despite limited labeled samples.

## 1. Introduction

The integration of industrial robots into modern manufacturing highlights the importance of timely and accurate fault detection. Their seamless functioning is essential for the production processes, since equipment failures leads to costly downtime and unnecessary maintenance [1,2]. Achieving reliable fault diagnosis when labeled data are scarce is a major challenge for the industry. This research gap motivates the development of fault diagnosis techniques for industrial robots, which are important for improving production efficiency and minimizing operational and maintenance costs.

With the widespread application of industrial robots in manufacturing and automation domains, the diagnosis and maintenance of their equipment malfunctions have become critical [1,2]. The seamless operation of robot systems is pivotal to the smooth progression of production processes, with equipment failures potentially leading to production stoppages and unwarranted maintenance costs [3,4]. Currently, there exists a challenge in achieving accurate fault diagnosis when labeled data are insufficient. Thus, researching industrial robot fault diagnosis techniques holds significant importance for enhancing production efficiency and reducing operational and maintenance expenses.

Recently, deep learning has been highlighted as a prevailing technique in fault diagnosis modeling [5]. It has gained prominence for its effectiveness in extracting intricate features and modeling complex patterns within industrial systems [6]. However, one significant hurdle with these models lies in their requirement of a large volume of data samples to achieve efficient training and reliable predictions [7,8]. When it comes to the specific domain of industrial robot fault diagnosis, collecting faulty data becomes particularly difficult. The infrequency of faults, combined with safety considerations and potential disruptions to manufacturing processes, makes it challenging to gather sufficient quantities of diverse and representative faulty data for robust deep learning applications. This scarcity of data often constrains the utility and performance of deep learning algorithms in practical scenarios involving industrial robot fault diagnosis. Consequently, there is a pressing need to explore diagnostic methods that can handle limited labeled data while maintaining high accuracy.

Various approaches have been developed to address fault diagnosis under limited data conditions [9,10]. Among these approaches, semi-supervised learning stands out for its ability to leverage both labeled and unlabeled data, thereby reducing the dependency on large volumes of annotated examples [11,12]. This technique combines the advantages of supervised learning with the potential of exploiting vast amounts of readily available yet untagged data, making it particularly suited for scenarios where acquiring fully-labeled instances is costly or impractical.

In the context of time series data analysis, which is prevalent in industrial robot monitoring and diagnostics, specific models like the Informer have shown remarkable effectiveness [13,14]. The Informer, designed specifically for long-sequence time series forecasting, excels at capturing temporal dependencies and patterns within the data. Integrating such an advanced time series modeling algorithm into a semi-supervised learning framework can enhance the accuracy of fault diagnosis in industrial robots. By harnessing both labeled fault cases and abundant unlabeled operational sequences, this combination could improve the compound fault diagnosis performance for industrial robotics systems.

This paper addresses the challenges of limited labeled data and the need for accurate fault diagnosis in industrial robots by proposing a semi-supervised learning-based approach. The foundation of our method is to leverage the accuracy of fault diagnosis of industrial robots through semi-supervised learning, which can make full use of labeled and unlabeled data. Semi-supervised learning enhances the learning process by inferring patterns from the vast majority of unlabeled data. Hence, it can improve the model’s performance to generalize and identify faulty patterns.

The main contribution of this work is threefold: (1) we harness the Informer’s ability to effectively capture long-term dependencies in time series data, enhancing the diagnostic precision by discerning subtle patterns and anomalies that are characteristic of the robot’s operation. (2) The proposed semi-supervised framework mitigates the reliance on labeled data alone, enabling it to automatically learn and exploit knowledge contained within unlabeled instances, thereby improving the overall fault diagnosis capability. (3) An experimental study based on the robotic fault dataset is performed to demonstrate the merits of the proposed semi-supervised Informer algorithm. By applying this method to industrial robots, this study demonstrates its efficacy in accurately diagnosing both single and compound faults, offering a promising direction for future advancements in industrial robotics maintenance and reliability. The rest of this paper is organized as follows: Section 2 reviews the latest advances in semi-supervised learning and deep learning in fault diagnosis. Section 3 reports the details of the proposed approach. Section 4 demonstrates an experimental study, and Section 5 draws the conclusion.

## 2. Literature Review

### 2.1. Recent Advances in Fault Diagnosis

In recent years, the field of fault diagnosis has seen remarkable strides, driven by advanced sensing technologies and data analytics techniques. Researchers have devoted substantial efforts to developing cutting-edge methods that can promptly and accurately detect and classify malfunctions in complex mechanical and electrical systems [6,15,16]. The advance of deep learning, particularly in its application to time series data and signal processing, has revolutionized the way faults are diagnosed, transcending traditional rule-based or statistical approaches [17,18].

Fault diagnosis has been studied for decades. To address the challenges posed by emerging unknown defects that conventional methods often fail to identify, our study introduces a groundbreaking approach rooted in incremental learning, Zhao et al. [19] introduced an incremental learning approach for the real-time identification of previously unseen wafer defects. Li et al. [20] introduced an unsupervised continuous passive network for machine failure diagnosis under different working conditions. Fault diagnosis is an important task in the industry. With advanced sensing and data analytics techniques, researchers have explored new approaches to detecting and diagnosing faults in complex mechatronic systems. One of the popular approaches is the deployment of machine learning algorithms, particularly deep learning methods such as convolutional neural networks (CNNs). CNNs have demonstrated their advantage in image recognition tasks and have been adapted for fault diagnosis applications due to their ability to extract features from raw sensor data. Eren et al. [21] combined a sensor fault diagnosis algorithm based on CNN with training samples to train the neural network and test its accuracy using test samples. These efforts have led to improved diagnostic accuracy and reduced downtime costs for affected systems. Zhang et al. [22] introduced a CNN enhanced with an attention mechanism, specifically the Convolutional Block Attention Module (CBAM-CNN), for diagnosing bearing faults. This methodology utilizes the CBAM to augment the network’s ability to extract fault features in the time-frequency domain. Additionally, the approach incorporates a visualization tool known as Gradient-weighted Class Activation Mapping (Grad-CAM), which improves the CNN’s interpretability by producing heatmaps on the fault’s time-frequency representations. To tackle the challenge of acquiring high-quality motor signals in complex noisy environments, Huang et al. [23] proposed a fault diagnosis model combining an attention mechanism, the AdaBoost method, and a wavelet noise reduction network. Firstly, multiple wavelet bases, soft thresholding, and index soft filters are optimized to train diverse wavelet noise reduction networks capable of restoring signals under various noise conditions. Secondly, a CNN classification module is integrated to build end-to-end classification models that can accurately identify faults.

Recently, transformers have been prevailing in the field of fault diagnosis modeling. Transformers can process the monitoring data in complex systems and detect patterns of faults. By modifying the transformer architectures, researchers have developed variants for fault diagnosis, leading to improved diagnostic accuracy and faster detection times. These advancements enable proactive maintenance, reducing the risk of catastrophic failures and minimizing downtime costs. Xiao et al. [1] proposed a Bayesian Variational Transformer (Bayesformer) to address the issues mentioned above. Unlike previous studies, Bayesformer treats all attention weights as latent random variables rather than fixed values, which enables an ensemble of networks to be trained, improving the model’s generalization capabilities. Chen et al. [3] introduced a dual transformer approach for the denoising of monitoring data and fault diagnosis of industrial robots. A compact Uformer was designed for the denoising of the monitoring data, and a compact convolutional transformer was developed for the fault diagnosis modeling. Gong et al. [24] developed a novel framework that integrates a Hierarchical Vision Transformer (NHVT) with a Wavelet Time-Frequency Architecture and a Multi-Source Information Fusion (MSIF) strategy to improve stability through the extraction and integration of diverse features. Initially, multi-source signals are transformed into two-dimensional representations of time and frequency. The NHVT is then employed to amplify the nonlinear characteristics of these feature maps, thereby enhancing the fault features. Following this, the diagrams representing multi-source information are consolidated into the NHVT to generate more detailed representations. Finally, the effectiveness of the NHVT is demonstrated using two separate multi-source datasets.

It is evident from the existing research that a significant portion of the academic community has dedicated efforts to the advancement of methodologies aimed at precisely detecting malfunctions within intricate mechanical and electrical systems. The advancement of deep learning, with a particular emphasis on its utilization for analyzing time series data, has revolutionized conventional diagnostic methodologies. This shift has led to improvements in diagnostic accuracy and efficiency, surpassing the capabilities of traditional statistical methods and conventional machine learning techniques. The integration of deep learning algorithms has not only enhanced the sensitivity and specificity of fault diagnosis, but has also facilitated the extraction of more nuanced patterns and relationships within the data, thereby offering a more robust and reliable framework for system health monitoring and maintenance.

### 2.2. The Latest Research on Few-Shot Learning in Fault Diagnosis

Recent advances in few-shot learning have opened up new possibilities for fault diagnosis in complex systems. This emerging technology enables efficient and effective identification of faults with minimal labeled data, making it particularly useful in situations where access to extensive labeled data is limited. Few-shot learning approaches such as semi-supervised learning, meta learning, and transfer learning aim to generalize knowledge from a small number of labeled examples to unseen data, allowing for rapid and accurate fault detection and diagnosis.

Semi-supervised learning is a type of effective approach to alleviating data imbalance issues. Shen et al. [25] reported a semi-supervised learning approach for bearing fault diagnosis. To extract local and global features, multi-scale dilated convolution squeeze-and-excitation residual blocks were designed. A classifier generative adversarial network was adopted for multi-task learning, which combines unsupervised and supervised learning to enhance the generalization ability. Supervised learning is then applied to refine the final model, which extracts multi-scale features and benefits from implicit data augmentation. Yu et al. [12] proposed an approach for intelligent bearing fault diagnosis when the labeled data are limited. The approach uses data augmentation and metric learning in three stages. In the first stage, seven data augmentation strategies were used to expand the feature space for limited labeled samples under each healthy condition. An optimization objective combining cross entropy loss and triplet loss was adopted to increase the margin between feature distributions of limited labeled samples under different healthy conditions. In the second stage, K-means clustering was used to obtain cluster centers for limited labeled samples under different healthy conditions. In the third stage, label information for unlabeled samples was estimated based on the membership between feature spaces. Zhang et al. [2] combined information stream fusion with semi-supervised learning techniques. Their approach employs a coupled convolutional residual network to merge information streams, with vibration and acoustic emission signals acting as the primary inputs. The features derived from these two streams are combined through concatenation to enhance the feature fusion. Moreover, they introduced a semi-supervised learning strategy that leverages labeled, accurately predicted, and unlabeled data to boost diagnostic precision.

Besides semi-supervised learning, meta-learning has received increasing attention in fault diagnosis. Luo et al. [26] introduced a meta-learning strategy based on the Elastic Prototypical Network for few-shot fault diagnosis in environments characterized by variable speeds, closely mirroring practical conditions. This approach incorporates a reinforced feature encoder enhanced with a squeeze-and-excitation attention mechanism to distill pertinent features from intricate signals under fluctuating speeds. Additionally, an elastic measurer is integrated, featuring an adjustable factor to facilitate adaptable classification across various fault categories. Shao et al. [27] developed a methodology grounded in Task-Supervised Almost No Inner Loop, which refines the internal loop’s backbone via a residual network to bolster feature reuse in uncharted domains. They incorporate an auxiliary component that formulates a task-adaptive loss function, enabling the adjustment of the inner loop meta-learner’s weight parameters based on the performance across multiple meta-diagnostic tasks.

Existing research in fault diagnosis often relies heavily on having a large amount of labeled data to train deep learning models. However, in the case of industrial robots, obtaining enough labeled data on equipment failures is difficult and crucial for keeping factories running smoothly. The existing methods help to diagnose issues, but still face the significant problem of needing a large amount of labeled data. To address this challenge, we propose a semi-supervised learning technique with Informer, which is great at understanding patterns in time series data. The Informer helps to find both short-term and long-term patterns that can signal a problem. By combining labeled and unlabeled data in this semi-supervised way, the method reduces the need for many labeled examples. It uses all available data to learn more effectively and make more precise diagnoses, even for complex, compound faults in industrial robots.

## 3. Methodology

For the composite fault diagnosis of industrial robots, the semi-supervised Informer model approach first involves systematically collecting online monitoring data from the robot system, which usually includes a large number of unlabeled instances and a small number of labeled instances, reflecting the scarcity of labeled data in industrial settings. For these data, data cleaning is first performed to remove outliers and noise caused by sensor errors or operational errors. Subsequently, data normalization processes such as min–max normalization or Z-score normalization are implemented to ensure that all feature values are on the same scale for efficient model training. In addition, feature engineering is required, including the extraction of statistical features, frequency domain features, or the construction of multi-dimensional time window features to enhance the predictive ability of the model. The built Informer model adopts a multi-head self-attention mechanism and integrates probabilistic sparse attention technology and convolutional layers, all of which are designed to optimize the processing efficiency and accuracy of long-sequence data. The model also includes fully connected layers and adaptive global–local attention distillation technology, which is particularly suitable for capturing long-term dependencies and key patterns in time series data, thereby effectively improving the ability to predict complex robot operation failures. This comprehensive approach makes full use of modern deep learning technology to provide an accurate and practical solution for industrial robot fault diagnosis.

**Figure 1:**
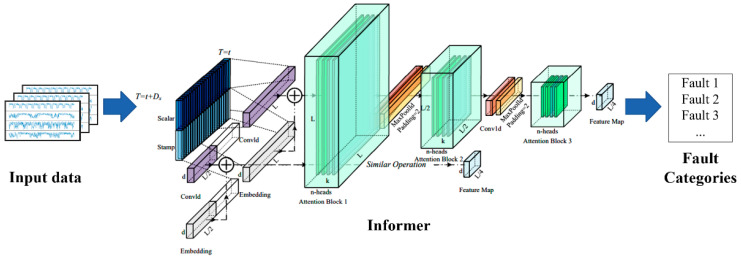
The structure of the Informer network.

Subsequently, a semi-supervised learning strategy is used where the Informer model is trained on the limited labeled data and the abundant unlabeled samples. The label propagation algorithm enables the diffusion of label information from known instances to unlabeled ones, thus enhancing the model’s generalization capabilities even under conditions of imbalanced or scarce labeled data availability. The model’s diagnostic accuracy is assessed using an independent test dataset containing both simple and compound fault scenarios. Finally, following successful validation, the semi-supervised Informer model, optimized through gradient descent-based algorithms like Adam, is prepared for deployment in actual industrial environments, offering accurate and efficient fault diagnosis for maintaining optimal operational efficiency and reducing the unnecessary downtime and maintenance costs associated with robot malfunctions. The overall flow of the proposed approach is demonstrated in Figure 1.

### 3.1. Informer-Based Fault Diagnosis

**Figure 2:**
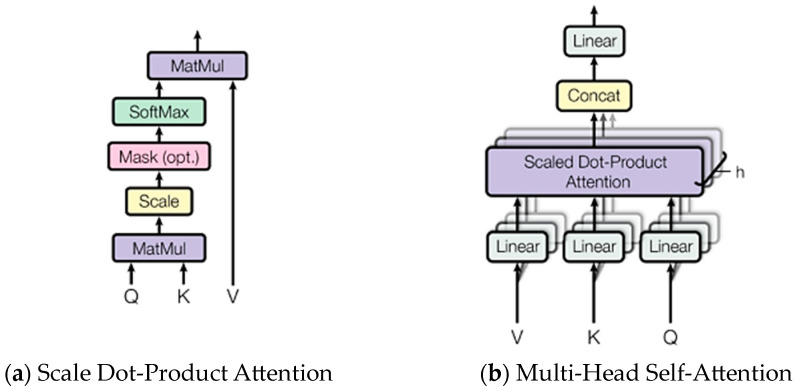
The structure of scale dot-product attention and multi-head self-attention.

The Informer is an effective architecture tailored for multi-dimensional time series forecasting and comprises a suite of interconnected components that work cooperatively to enhance prediction accuracy. The multi-dimensional time series are firstly processed using the time window approach, and are then input into the Informer. The important parts of the encoding module, which harnesses masked multi-head self-attention mechanisms, include convolutional layers, fully connected networks, and probabilistic sparse attention and adaptive global–local distillation techniques. The encoder’s structure is designed in a tiered manner, with several stacked layers as depicted in Figure 2, where each layer integrates both a multi-headed self-attention mechanism and a feed-forward network.

The multi-head self-attention submodule serves to identify and capture intricate dependencies between various positions within the input sequence, effectively extracting discriminative features at every step. It is the core part of the Informer model. The multi-head self-attention mechanism is an extension of the attention mechanism, which allows the model to learn information from different representation subspaces simultaneously. Given an input X, the matrices Q, K, and V can be obtained by the linear transform, which can be expressed as:

(1) $$Q=XW^{q}$$

(2) $$K=XW^{k}$$

(3) $$V=XW^{v}$$

where $W^{q},W^{k},\;\;and\;W^{v}$ are the matrices of the learnable parameters. 

Then, the obtained matrices are further fed into the scaled dot product attention to obtain the attention score, which can be expressed as follows:

(4) $${Head\_Score}_{1}=\frac{softmax(Q\times K^{T})\times V}{\sqrt{d}}$$

where d is a scalable coefficient.

In multi-head self-attention, Q, K, and V are linearly transformed h times to different representation spaces, and an attention operation is performed on each representation space. The structure of scale dot-product attention and multi-head self-attention are demonstrated in Figure 2. Finally, the outputs of these attention operations are concatenated and the final output is obtained by linear transformation again. Each attention head can be expressed by the following formula:

(5) $${head}_{i}=Attention\left(QW_{i}^{Q},KW_{i}^{K},VW_{i}^{V}\right),i=1,\ldots ,N$$

where $W_{i}^{Q},W_{i}^{K},\;and\;W_{i}^{V}$ are the matrices of the learnable parameters.

This submodule ensures that each position receives a unique representation informed by its context within the sequence. A critical aspect of the Informer’s design is its use of masking within the masked multi-head self-attention component, which makes sure the model can defend against lookahead bias. Individual attention heads are strategically masked to restrict access to future information during the prediction process, thereby preserving temporal causality and enhancing model reliability for accurate fault diagnosis tasks in industrial robotics. Its attention distribution conforms to a long-tail pattern, where only a select few dot-product calculations significantly contribute to the focus on key fault features, while the majority of other dot products exert minimal influence that can be safely disregarded. Based on such results, the *i*th query’s attention is introduced:

(6) $$C(h_{i},L,Q)=\sum_{j}\frac{l(h_{i},l_{j})}{\sum_{a}l(h_{i},l_{a})}q_{j}=E_{b(l_{j}|h_{i})}[q_{j}]$$

To quantify the sparsity of the attention given to the ith query in the Informer model, we utilize the Kullback–Leibler (KL) divergence metric. This involves measuring the dissimilarity between the attention distribution concentrated on fault features and a uniform distribution. The larger the calculated KL divergence value, the greater the disparity between the actual attention pattern for the fault features and an evenly distributed one, which implies that the difference is more pronounced and suggests a higher probability of the distribution being unimodal—hence indicating increased sparsity. Therefore, by comparing the resulting KL divergence with that expected from a uniform distribution, it becomes evident whether the model’s focus on fault features exhibits a sparse or dense concentration of attention. The attention score of each key and the top u dominant queries can be calculated as follows:

(7) $$R(H,L,Q)=Soft\max(\frac{\bar{H}L^{T}}{\sqrt{b}})Q$$

The feedforward network integrated within the Informer’s encoder structure is a composite of several computational layers, including convolutional layers, exponential linear unit (ELU) activation functions, max pooling operations, and fully connected layers. These components collectively serve to refine and distill important feature representations from the input data. Convolutional layers play a pivotal role by decreasing the dimensionality of features while preserving spatial relationships, which not only contributes to reducing the model’s parameter count, but also enhances computational efficiency. The filters in these layers capture local patterns and structures in the data. Max pooling layers further condense information by selecting the maximum values within specified regions, thereby highlighting the most salient features and promoting spatial invariance. Finally, the fully connected layers act as an integral part of this process by performing non-linear transformations on the feature maps generated in the convolutional stage. They are capable of learning intricate associations between various levels of extracted features, allowing the model to discern complex dependencies that might underlie the diagnostic signals in industrial robot systems. The specific formula is shown as follows:

(8) $$Y_{i+1}^{t}=MaxPool(ELU(Conv1d([Y_{i}^{t}{]}_{AB}))$$

The adaptive global and local self-attention distillation mechanism embedded within the Informer model facilitates the learning of intricate associations between various positions in the input sequence, as well as the interdependencies among different attention layers. This advanced capability enables the model to effectively adapt to sequences of varying lengths, thereby enhancing its predictive prowess. By refining its understanding of these relationships, the Informer is better poised to capture both the global context and local details, which results in improved feature representation and a heightened resilience against potential interference from irrelevant or noisy data points. The distillation process optimizes information transfer across layers, ensuring that the distilled knowledge contributes to more accurate and robust fault diagnosis in industrial robotics applications.

The adopted loss function for fault classification in this study is the widely used cross-entropy loss, a well-established choice in the context of identifying different fault types. This particular loss metric proves particularly effective for the task at hand, as it quantifies the discrepancy between the predicted probabilities and the actual class labels. Notably, cross-entropy loss demonstrates commendable robustness when dealing with imbalanced datasets, which are common in electromechanical equipment monitoring scenarios where certain fault classes may be underrepresented. Its ability to handle such data distributions ensures that the model remains sensitive to minority classes, thereby enhancing overall diagnostic accuracy and reliability. The cross-entropy loss can be expressed as:

(9) $$loss(x,y)=-\log(\frac{\exp(x[y])}{\sum_{j}\exp(x[j])})$$

The Informer algorithm has made innovative contributions in the form of probabilistic sparse attention and adaptive global local attention extraction. The former introduces a new mechanism that focuses on key positions in the input sequence by learning probability distributions, thereby directing attention computation towards sparsity. Compared with traditional autoregressive models, this significantly reduces computational complexity, enabling Informer to maintain efficient processing while still retaining its ability to capture long-range dependencies in extended sequences. The latter feature innovatively combines global attention and local attention, where global attention models the entire sequence while local attention focuses on specific segments and dynamically adjusts their relative weights through adaptive learning.

### 3.2. Label Propagation Algorithm

In traditional supervised learning, models require a significant amount of labeled data for training. However, in many real-world scenarios, obtaining large volumes of labeled data is time-consuming, costly, or simply not feasible. The label propagation (LP) algorithm bypasses this issue by using both labeled and unlabeled instances in the learning process. It does not discard any available data, but instead leverages the information contained in all samples, even those without explicit labels. LP is a graph-based semi-supervised learning technique that propagates labels from labeled data points to unlabeled ones by constructing an affinity graph where nodes represent data instances and edges reflect their similarity. The main idea is that neighboring nodes are more likely to share the same class label. The technical path of LP is shown as follows:

**Graph Construction:** Build an undirected weighted graph G = (V, E, W), where V represents the set of all nodes (labeled and unlabeled data samples), E represents the set of edges connecting nodes, and W is the adjacency matrix encoding the similarity between each pair of nodes.

**Initialization:** Assign initial labels to the labeled subset of nodes (usually determined by ground truth labels). All unlabeled nodes start with no definitive label or sometimes with a uniform distribution over possible classes.

**Label Propagation Iteration:** For each iteration t: Update the label vector $L_{j}^{\left(t\right)}$ for each node i using its neighbors’ current label distributions:

(10) $$L_{i}^{\left(t+1\right)}=\frac{\sum_{j\in N(i)}w_{ij}L_{j}^{\left(t\right)}}{\sum_{j\in N(i)}w_{ij}}$$

where N(i) is the neighborhood of node i and $w_{ij}$ is the weight of the edge between nodes i and j.

**Convergence Check:** Continue iterating until the label distribution converges, meaning the difference between $L_{i}^{\left(t\right)}$ and $L_{i}^{\left(t-1\right)}$ falls below a predefined threshold or reaches a maximum number of iterations.

**Final Label Assignment:** After convergence, assign the most probable label to each node based on its final label distribution:

(11) $$L_{i}^{\left(t+1\right)}=(1-\alpha )L_{i}^{\left(t\right)}+\alpha \frac{\sum_{j\in N(i)}w_{ij}L_{j}^{\left(t\right)}}{\sum_{j\in N(i)}w_{ij}}$$

where α is a smoothing parameter balancing between the previous label assignment and the new propagated values.

## 4. Experimental Study

### 4.1. Dataset

To validate the performance of our proposed semi-supervised Informer approach, a compound fault injection experiment was performed on a six-axis industrial robot to gather faulty data. Within this experiment, we purposefully induced compound faults by substituting healthy components with malfunctioning reducers and motors retrieved from real-world manufacturing contexts. These substituted faulty parts demonstrated initial signs of problems, including low-level abnormal sounds and small-scale oil leaks.

**Figure 3:**
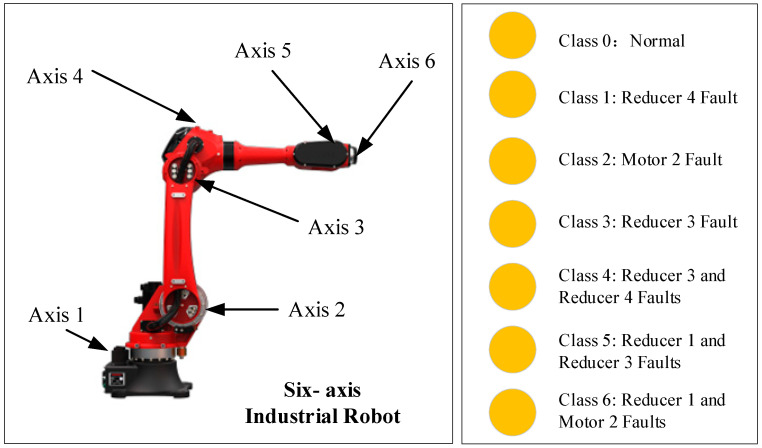
The demonstration of the dataset.

In the experiment, we collected the feedback current data from the motor drivers in the third axis of transmission system. Given that early-stage faults tend to display faint and hard-to-detect patterns, running the robot with these faulty components allowed for the recording of these elusive fault signatures. In the next stage, the three-phase current was carefully recorded using a built-in Hall sensor within the motor driver unit. We concentrated on the q-axis current data, which oscillated at a frequency of 1 Hz, for our modeling efforts. This experimental dataset was rich with detailed information about the robot’s health and performance under compounded fault conditions. The collected data are demonstrated in Figure 3.

In this study, we pre-processed the online monitoring data of industrial robots to ensure data quality and the applicability of model training. Firstly, in the data cleaning stage, statistical methods and machine learning algorithms were used to identify and handle outliers and missing values. Specifically, we calculated the z-score (standard score) for each data point, which is the distance of the data point from the mean in standard deviation. Typically, z-scores greater than 3 in absolute value are considered as outliers. Once the outliers were identified, they were replaced by the mean value of the last three data points. In this study, there approximately 30 outliers were identified and replaced. In the dataset, there were approximatively 230 missing values. For the missing values, it can be seen that over 98% the missing values were not continuous, and were then imputed by the mean value of the last three data points. For the remaining 2% of the missing values, which were continuous, it was difficult to accurately impute the missing points due to the high uncertainty of imputation. Hence, those continuous missing values were removed. In the next stage, we performed Z-score normalization to give all features a zero mean and unit variance, eliminate dimensional and scale differences, and enhance the model’s training efficiency and generalization ability. In the next stage, we utilized a sliding window approach to the time series data to obtain the 3D data input for the deep learning algorithms. Our preprocessing process was integrated with deep learning models into a data flow processing system. The data that underwent cleaning and standardization processing were transmitted in real time to the model training environment through a specially designed data pipeline. This pipeline could automatically process new incoming monitoring data, ensuring continuous learning and updating of the model. 

In this study, we utilized detailed statistics and reasonable allocation of the sample data involved in the composite fault diagnosis task of industrial robots. Table 1 presents the distribution of labeled and unlabeled data across different fault categories in our experimental dataset. The dataset was prepared to ensure a comprehensive representation of various fault scenarios in industrial robots, with a focus on reducers and motors. The labeled data were randomly selected from the original dataset. As for the rest of the data samples in the original dataset, the labels were removed and used as unlabeled data.

When constructing the training and testing sets, we followed the typical machine learning data-partitioning principle of retaining a certain proportion of data as independent test sets to evaluate the model’s generalization ability. We adopted fivefold cross-validation. We adopted fivefold cross-validation to ensure the robustness and reliability of our results. Fivefold cross-validation divides the training dataset into five equal parts. During the cross-validation process, the model was trained and tested five times. Each training and testing process used a different fold as the validation set, while the remaining four folds were used for training. This method allowed us to evaluate the model’s performance across different parts of the dataset. All unlabeled samples were included in the training set for information dissemination and model optimization in the semi-supervised learning process. These samples were evenly distributed across all categories to be identified, ensuring a comprehensive test of the testing set’s ability to diagnose various types of faults.

### 4.2. Experimental Setup

The parameter setting was essential. In the Informer model settings, we utilized a specific configuration to optimize the performance for industrial robot fault diagnosis. We chose a model dimension of 512 and employed eight attention heads in the multi-head self-attention layer to enable the model to attend to different parts of the input data simultaneously. The encoder layers were configured in a tiered manner with three layers, two layers, and one layer, respectively, to progressively extract higher-level features. To handle the complexity of the relationships within the data, we employed a feed-forward network with a hidden layer size of 512. We adopted probabilistic sparse attention to focus the model’s attention on key positions in the input sequence, which is particularly effective for time series data. The embedding type was set to fixed, as we did not require learnable embeddings in our application. 

In the experimental setup regarding the Informer model, the cross-entropy loss function and Adam optimization algorithm were adopted. Cross-entropy, being a commonly adopted loss function in classification tasks, is instrumental due to its ability to directly measure the dissimilarity between predicted values and true labels, thus facilitating the rapid and effective training of the model. Its advantageous mathematical properties help to mitigate the vanishing gradient problem common in complex architectures such as deep neural networks. Moreover, when dealing with multi-classification scenarios, cross-entropy loss inherently focuses the model’s attention on misclassified samples, thereby enhancing the model’s generalization performance. In the specific implementation of the Informer model for the benchmarking experiments, the learning rate was set to 0.0001, a value often conducive to stable and steady improvement without causing erratic oscillations or premature convergence. The number of iterations was configured to be 100, ensuring ample opportunities for the model to learn from the data and refine its predictions.

In the benchmarking experiments, the setting for the Informer model and the introduction of comparative algorithms were outlined. Initially, the configuration details for the Informer model were presented, with the parameters adjusted to suit the specific requirements of compound fault diagnosis in the industrial robot context. Presently, the benchmarking algorithms will be introduced, which include:

**Convolutional Neural Networks (CNNs):** A 1D-CNN is designed for time series modeling. It has the inherent ability to detect local dependencies and spatial features within the experimental setup. We designed a four-layer 1D-CNN network with the number of nodes set at 128.

**Long Short-Term Memory (LSTM) Networks:** Similarly, LSTMs, another prominent RNN variant, are passively implemented to gauge their capacity to diagnose compound faults within industrial robots based on historical data. We designed a LSTM network with four layers, and the number of nodes was set at 128.

**Vanilla Transformers:** Following their notable success in natural language processing, vanilla transformers are passively incorporated into the experimental design to assess their applicability and performance in time series analysis for the industrial robot scenario, focusing on their capability to recognize global dependencies across input sequences. The hidden dimension of vanilla transformers was set at 128 and the number of transformer blocks was set at two.

To evaluate the performance of the fault diagnosis model, we used precision, recall, and F1 score as evaluation metrics. Precision measures the fraction of true positive predictions out of all positive predictions made by the model. It quantifies the ability of the model to avoid false positive errors, where instances that are actually healthy are misclassified as faulty. Recall, meanwhile, is a measure of the fraction of true positive predictions out of all actual positive examples in a dataset. It can quantify the ability of the model to detect actual failures, avoiding false positives where instances are misclassified as healthy when they fail. The F1 score is the harmonic mean of precision and recall, providing a single metric that balances the trade-off between the two, which is useful when the class distribution in the dataset is imbalanced. The F1 score is often used in fault diagnosis scenarios.

### 4.3. Experimental Results

**Figure 4:**
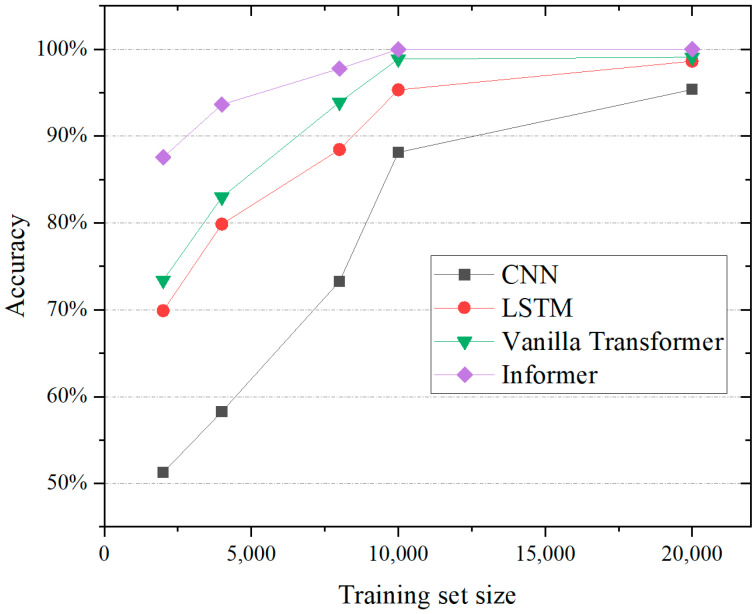
Comparison of different algorithms modeled using datasets of different sizes.

The performance of the semi-supervised Informer model was then compared against these benchmark algorithms to objectively demonstrate its potential superiority and improved effectiveness in accurately diagnosing compound faults in industrial robots under different data availability scenarios. The results can be seen in Figure 4. A series of experiments was conducted by varying the quantity of labeled data available for training. This strategy aimed to examine the model’s adaptability and robustness in scenarios where labeled data are limited, simulating real-world conditions, where manual labeling can be expensive and time-consuming. The experiments involved systematically reducing the proportion of labeled data, ranging from a fully supervised setting with a considerable number of labeled instances to a sparse-label regime with a minimal quantity. For each level of labeled data availability, the model was trained and tested. As the size of the training set increased, the accuracy of all four models improved. However, the rate at which the accuracy improved varied between the models. The Informer model exhibited the most significant improvement in accuracy with increasing training set size, achieving the highest accuracy overall. In contrast, the vanilla transformer model showed the least improvement in accuracy and remained the least accurate of the four models throughout. The LSTM and CNN models showed similar performances, with the LSTM slightly outperforming the CNN.

**Figure 5:**
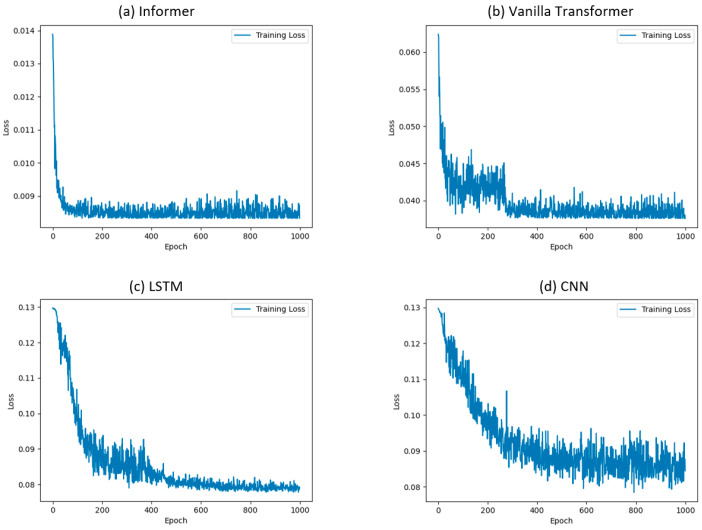
The loss curves of different algorithms modeled using a full dataset.

The loss curve of all the algorithms when the full dataset was used is plotted in Figure 5. It can be seen that Informer converged when the epoch reached 200, which was faster than the other benchmarking algorithms.

**Figure 6:**
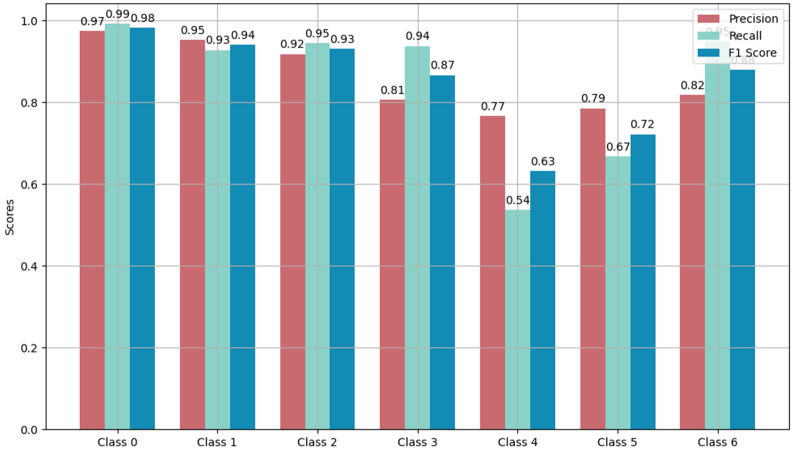
The precision, recall, and F1 score of Informer modeling using the full training dataset.

Figure 6 provides detailed insights into the performance of the Informer model on various classes within a dataset. Class 0 stood out as having the best precision, recall, and F1 scores, indicating that the model accurately identified this class more effectively compared to other classes. On the other hand, Class 4 displayed the poorest performance, with lower precision, recall, and F1 scores. This suggests that the model struggled to identify instances belonging to Class 4 correctly or may have misclassified them as other classes. In general, the model’s performance varied significantly across different classes. Some classes exhibited high accuracy, such as Classes 0, 3, and 6, while others, like Classes 1, 5, and especially 4, showed relatively poor performances. This disparity highlights the need for further improvements in the models’ abilities to handle certain classes more effectively.

**Figure 7:**
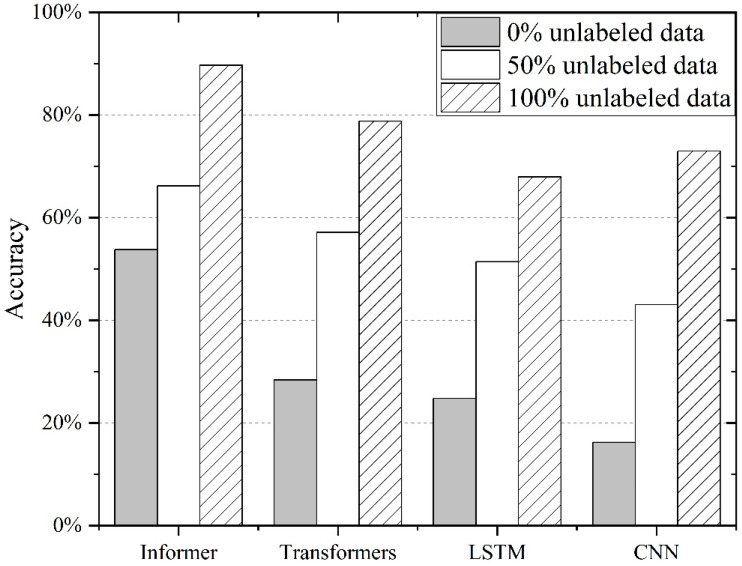
The results of semi-supervised learning for different algorithms.

Figure 7 illustrates the effect of incorporating different percentages of unlabeled data on the accuracy levels of different algorithms. When there were no unlabeled data present (0%), the Informer model still achieved the highest accuracy, followed closely by the transformer model. The LSTM and CNN models performed less well, with lower accuracy scores. As the amount of unlabeled data increased to 50%, all four models showed improvements in accuracy. The Informer continued to lead with the highest accuracy, while the transformer, LSTM, and CNN models also showed increased performance. Finally, when 100% of the data were unlabeled, the Informer maintained its position as the top-performing model, with the highest accuracy. The transformer, LSTM, and CNN models continued to improve in accuracy as well, but they remained below the Informer’s level of performance.

**Figure 8:**
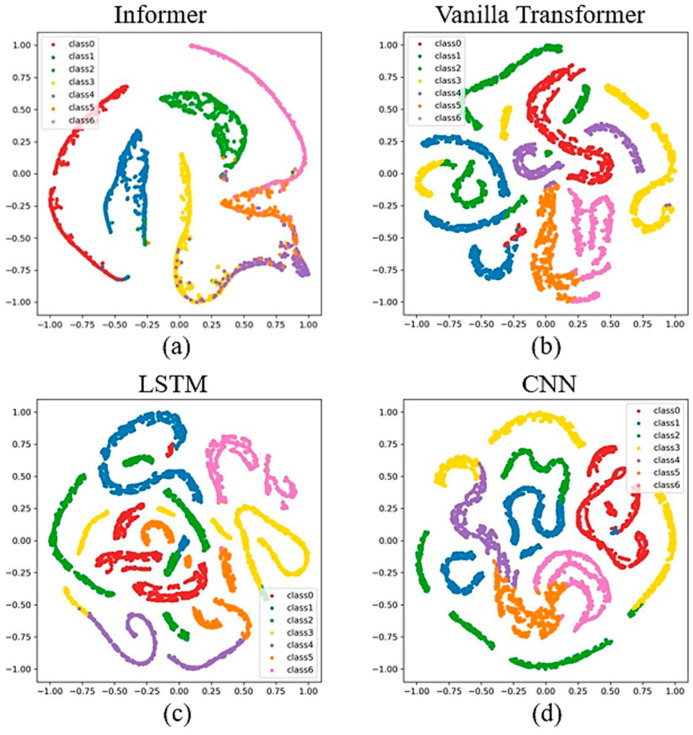
The T-SNE of semi-supervised learning for different algorithms with 100% unlabeled data.

Figure 8 depicts the results of applying different models to classify faults in a system where the data consist of multiple fault categories and normal conditions. The semi-supervised Informer model, as shown in Figure 8a, was able to effectively distinguish between normal and faulty states, with the normal state represented by “class 0” being highly clustered in the feature space, clearly separated from the faulty states. For single-fault types, the model also performed well in classification, although slight overlaps could be observed in some boundary regions. However, when it comes to composite faults, the t-SNE results indicate a certain degree of inter-class mixing, particularly between “class 4” and “class 5”, which could reflect the similarity in feature representation and the complexity involved in diagnosing these composite faults. On the other hand, the vanilla transformer, LSTM, and CNN models, as depicted in Figure 8b–d, respectively, showed blurred boundaries between the normal class and the fault classes, resulting in relatively poorer classification performance compared to the semi-supervised Informer.

**Figure 9:**
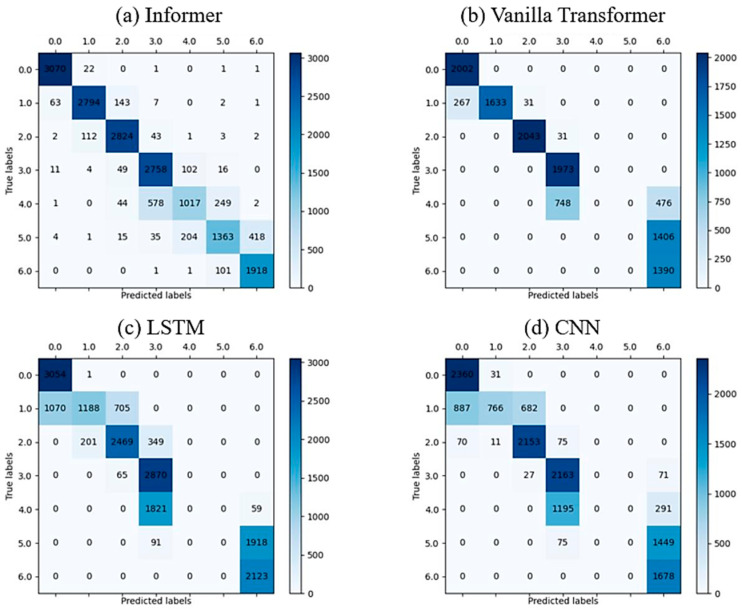
The confusion matrix of semi-supervised learning for different algorithms with 100% unlabeled data.

The confusion matrices for the Informer, vanilla transformer, LSTM, and CNN models are presented in subplots (a), (b), (c), and (d) of Figure 9, respectively. The Informer model (subplot (a)) showed high accuracy in classifying the samples, with most true labels matching the predicted labels. There were only a few misclassifications, mainly between classes 1 and 2 and classes 3 and 4. The vanilla transformer (subplot (b)) also showed reasonable accuracy, though with more misclassifications, particularly between classes 1 and 2 and classes 3 and 4. Subplots (c) and (d) showed the confusion matrices for the LSTM and CNN models, respectively. Both models exhibited more errors in their predictions, with LSTM making more mistakes between classes 1 and 2 and classes 3 and 4, while CNN made more errors across all classes. Overall, the Informer model appeared to perform better in terms of accuracy, with fewer misclassifications.

**Figure 10:**
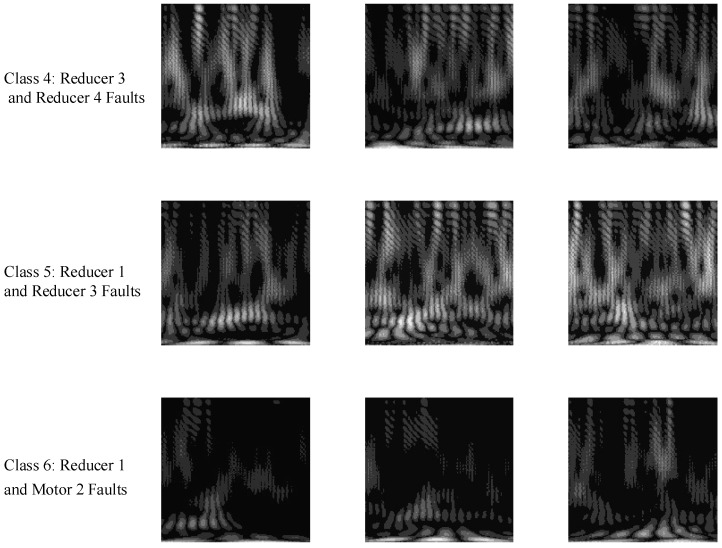
The CWT images of class 4, 5, and 6.

It can be seen from the confusion matrix that the samples of classes 4, 5, and 6 were difficult to correctly classify. In order to further investigate the reason that led to the misclassification, continuous wavelet transform was introduced to transform the data into time-frequency images. Morlet kernel was adopted and the data window for each transformation was set to 256. The randomly selected time-frequency images of classes 4, 5, and 6 are demonstrated in Figure 10. The time-frequency images indicate that the data in these three classes were highly similar, which impeded the correct classification of the deep learning models.

## 5. Discussion

During the benchmarking experiments, the Informer model was fine-tuned and tested against well-established algorithms such as CNN, LSTM networks, and vanilla transformers. These comparative algorithms represent prevalent approaches in time series analysis and pattern recognition for fault diagnosis. However, the semi-supervised Informer showed superior adaptability and robustness in handling scenarios with limited labeled data, reflecting its core advantage over purely supervised methods. The results revealed that, under various levels of labeled data availability, the semi-supervised Informer consistently outperformed the benchmark models in terms of accurately diagnosing compound faults. Even with significantly fewer labeled samples, the model could converge to a stable and effective diagnosis, indicating its strong capacity to learn from unlabeled data and leverage this knowledge to enhance its performance.

In the experimental results, it can be seen that the classification of Classes 3 and 4 was challenging. Class 3 represents the single fault of reducer 3. In our experiments, we observed that this class was occasionally misclassified as class 1, which corresponds to the fault of reducer 1. The misclassification can be attributed to the subtle differences in fault patterns between reducer 1 and reducer 3. While reducer 1 and reducer 3 are different components of the industrial robot, their faults may exhibit similar characteristics, especially when the fault patterns are not distinctly different. This overlap in fault patterns makes it challenging for the model to accurately differentiate between these two classes, leading to some misclassifications. Class 4 represents the compound faults of reducer 3 and reducer 4. This class is more challenging to diagnose compared to single faults, as compound faults often present overlapping and complex patterns. In our experiments, we observed that class 4 was frequently misclassified as class 5, corresponding to the fault of reducer 4. This misclassification was likely due to the similarity in fault patterns between reducer 3 and reducer 4 when affected by compound faults. The overlapping patterns made it difficult for the model to distinguish between these two classes, resulting in some misclassifications. From the results of time-frequency images, it can be seen that the data in three compound fault categories were highly similar in terms of the time-frequency patterns. That is the reason why all the benchmarking deep learning models were unable to achieve accurate classification for the compound fault diagnosis. Informer achieved a compound fault diagnosis accuracy of 71.2%, which indicates its advantage in complex pattern learning. In order to further improve the compound fault diagnosis accuracy, it would be worthwhile to investigate new sensing techniques, such as vibration and feedback torque, that can effectively capture the compound fault patterns of industrial robots. 

Despite the results achieved with the semi-supervised Informer model for compound fault diagnosis in industrial robots, this study has certain limitations and points to avenues for future investigation. Firstly, the experiments were conducted with a specific industrial robot and subsystem. The model’s performance with limited labeled data is promising, but its scalability to other industrial robot types and subsystems has yet to be validated. Different robot brands, architectures, and sensors may introduce variations in the generated data, potentially impacting the model’s generalizability across multiple platforms. To address this issue, we will further expand our dataset to include a wide range of industrial robots, which will allow us to evaluate the model’s performance in real-world scenarios and make the adjustments to improve its generalizability. Secondly, the semi-supervised learning framework relies on the quality and diversity of the unlabeled data. To further enhance the model’s performance, future works will explore advanced clustering or active learning strategies to make better use of the unlabeled data, which will help semi-supervised learning to improve the model’s ability to learn from unlabeled data. Thirdly, integrating the Informer model with real-time monitoring systems presents challenges such as computational complexity and latency. To address these issues, we will streamline the model for faster inference without compromising diagnostic accuracy, which may involve model architecture modifications, optimization techniques, or the use of specialized hardware accelerators. Fourthly, while the semi-supervised Informer model is effective, there is a need for additional interpretability studies to provide clear explanations for its diagnostic decisions. This will increase trust among operators and maintenance personnel. We plan to incorporate explainable AI techniques, such as attention mechanisms or feature visualization, to clarify the reasoning behind the model’s outputs. This will promote safer and more informed decision-making processes in industrial environments, enhancing the overall reliability and safety of industrial robots.

## 6. Conclusions

In order to address the labeled data limitation issue in the compound fault diagnosis, this paper presents a semi-supervised learning approach employing an advanced Informer model for compound fault diagnosis in industrial robots. The proposed approach study combines the Informer’s inherent capability to capture long-term dependencies in time series data with the power of semi-supervised learning to enhance fault diagnosis performance. Through experimentation based on real-world data, the proposed method has been proven effective in accurately diagnosing both simple and complex faults in industrial robots, even when the number of labeled samples is insufficient. Comparative evaluation with established models like vanilla transformers, LSTM, and CNNs demonstrates the superiority of the semi-supervised Informer in producing clearer class separations and fewer misclassifications. The study acknowledges that further work is necessary to expand the model’s versatility across various industrial robotic systems and environmental conditions. In our future research, we will explore strategies to refine the selection and utilization of unlabeled data, extend the fault classification repertoire, and integrate the model into real-time monitoring frameworks with optimized computational requirements.

## Figures and Tables

**Table 1:** The number of labeled and unlabeled data in each class.

Fault Category	Labeled Samples	Unlabeled Samples
Class 0: Normal	2000	19,000
Class 1: Reducer 4 Fault	2000	10,000
Class 2: Motor 2 Fault	1800	19,000
Class 3: Reducer 3 Fault	1800	15,000
Class 4: Reducer 3 and Reducer 4 Faults	2000	12,000
Class 5: Reducer 1 and Reducer 3 Faults	1900	15,000
Class 6: Reducer 1 and Motor 2 Faults	2000	19,000

## Data Availability

The data presented in this study are available upon request from the corresponding author.

## Abbreviations

The following abbreviations are used in this manuscript:

CNNs	Convolutional Neural Networks
KL	Kullback–Leibler
ELU	Exponential Linear Unit
LSTM	Long Short-Term Memory
